# Analysis of distribution and antibiotic resistance of Gram-positive bacteria isolated from a tertiary-care hospital in southern China: an 8-year retrospective study

**DOI:** 10.3389/fmicb.2023.1220363

**Published:** 2023-09-28

**Authors:** Xiao Zhang, Liming Tan, Pengwen Ouyang, Haiyan Ma, Jianqiao Peng, Ting Shi, Liangyi Xie

**Affiliations:** ^1^Department of Clinical Laboratory, Hunan Provincial People's Hospital (The First Affiliated Hospital of Hunan Normal University), Changsha, China; ^2^Department of General Surgery, Hunan Provincial People's Hospital (The First Affiliated Hospital of Hunan Normal University), Ghangsha, China

**Keywords:** Gram-positive bacteria, distribution, drug resistance, epidemiology, surveillance

## Abstract

**Objective:**

Due to the severe drug resistance situation of Gram-negative bacteria, especially Gram-negative enterobacter, relatively little attention has been paid to the changes in Gram-positive bacteria species and drug resistance. Therefore, this study analyzed the prevalence and drug resistance of Gram-positive bacteria in a general tertiary-care hospital from 2014 to 2021, in order to discover the changes in Gram-positive bacteria distribution and drug resistance that cannot be easily identified, inform clinicians in their respective regions when selecting antimicrobial agents, and to provide the basis for the diagnosis of Gram-positive bacterial infection, and for the comprehensive and multi-pronged prevention and control of drug-resistant bacteria.

**Methods:**

A retrospective study was conducted on Gram-positive bacteria isolated from patients presented to a general tertiary-care hospital from January 2014 to December 2021. A total of 15,217 Gram-positive strains were analyzed.

**Results:**

During the 8-year period, the total number and the species of Gram-positive bacteria isolated from clinic increased continuously. The seven most common species were *Streptococcus pneumoniae* (21.2%), *Staphylococcus aureus* (15.9%), *Enterococcus faecium* (20.6%), *Enterococcus faecalis* (14.0%), and *Staphylococcus epidermidis* (7.8%), *Staphylococcus haemolyticus* (4.8%), *Streptococcus agalactiae* (3.6%). The isolation rates of *Staphylococcus aureus* and *Streptococcus agalactiae* increased, and the isolation rate of *Enterococcus faecium* decreased. The resistance rates of *Staphylococcus aureus* to erythromycin, clindamycin, tetracycline, rifampicin and furantoin decreased obviously. The resistance rates of *Streptococcus pneumoniae* to cefepime (non-meningitis) and ceftriaxone (meningitis) decreased significantly. The resistance rates of *Enterococcus faecium* to penicillin, ampicillin, erythromycin, levofloxacin, ciprofloxacin and furantoin rose rapidly from 50.3, 47.6, 71.5, 44.9, 52.3, and 37.5% in 2014 to 93.1, 91.6, 84.9, 86.8, 86.8, and 60.0% in 2021, respectively.

**Conclusion:**

The total number and the species of Gram-positive bacteria isolated during the 8-year period increased continuously. *Streptococcus pneumoniae* and *Staphylococcus aureus* are the main causes of positive bacterial infections in this hospital. The resistance rates of *Enterococcus faecium* to a variety of commonly used antibiotics increased significantly. Therefore, it is very important to monitor the distribution of bacteria and their resistance to antibiotics to timely evaluate and identify changes in drug resistance that are not easily detected.

## Introduction

The World Health Organization (WHO) has published a list of global priority pathogens-12 species of bacteria with critical, high, and medium antibiotic resistance. Among these pathogens, in addition to the drug-resistant Gram-negative bacilli that are under scrutiny, such as carbapenem-resistant *Acinetobacter baumannii, Pseudomonas aeruginosa* and Enterobacteriaceae, Gram-positive bacteria, particularly multidrug-resistant Gram-positive bacteria, such as methicillin-resistant *Staphylococcus aureus* (MRSA), vancomycin resistant *Enterococcus faecium* (VRE) and β-lactamase resistant *Streptococcus pneumoniae*, are also considered a major concern (Asokan et al., 2019).

The main resistance mechanisms identified include the alterations in the PBPs that lead to the destruction of antibiotic active sites; preventing the drug from reaching and binding to its target by modifying the bacterial structure, such as thickening peptidoglycan and altering the ribosomal structure, or overexpressing the drug efflux pump (Jubeh et al., 2020). Gram-positive bacteria exhibit a tremendous genetic capacity to acquire and develop resistance to almost all clinically available antibiotics. Therefore, its resistance to antibiotics must be treated as an evolving problem, and be monitored continuously to identify emerging resistance mechanisms to optimize antibiotic use and develop strategies to circumvent this problem. In this study, a retrospective study was conducted on Gram-positive bacteria isolated from a large tertiary hospital in southern China during the 8-year period from 2014 to 2021 to comprehensively analyze the characteristics of their distribution and changes in drug resistance, so as to provide evidence and ideas for the subsequent treatment of bacterial infections and antibiotic management.

## Materials and methods

### Source of strains

Pathogens were isolated from specimens that were collected from patients who had presented to a tertiary hospital from southern China during January 2014 to December 2021. A total of 15,217 Gram-positive strains were isolated and analyzed retrospectively.

### Bacteriological identification and drug sensitivity test

Species identification was performed using the VITEK-2 system (BioMérieux, Marcy l′Etoile, France), VITEK-MS system (BioMérieux, Marcy l′Etoile, France) was also used for the species identification since 2018. Antibiotic susceptibility testing was performed using the VITEK-2 system according to the recommendations proposed by the Clinical and Laboratory Standards Institute (CLSI).

### Statistical analysis

The Chi-square test was applied to analyze the demographic characteristics, Manner-Kendall test was applied to analyze the differences in proportions and trends between years, *Z*-value is used to assess the trend of drug resistance rates. The statistical analyses were performed using SPSS software, version 20.0 (IBM Corp, Armonk, NY, USA) and OriginPro software, version 2021 (OriginLab Corp, MA, USA).

## Results

**Figure 1:**
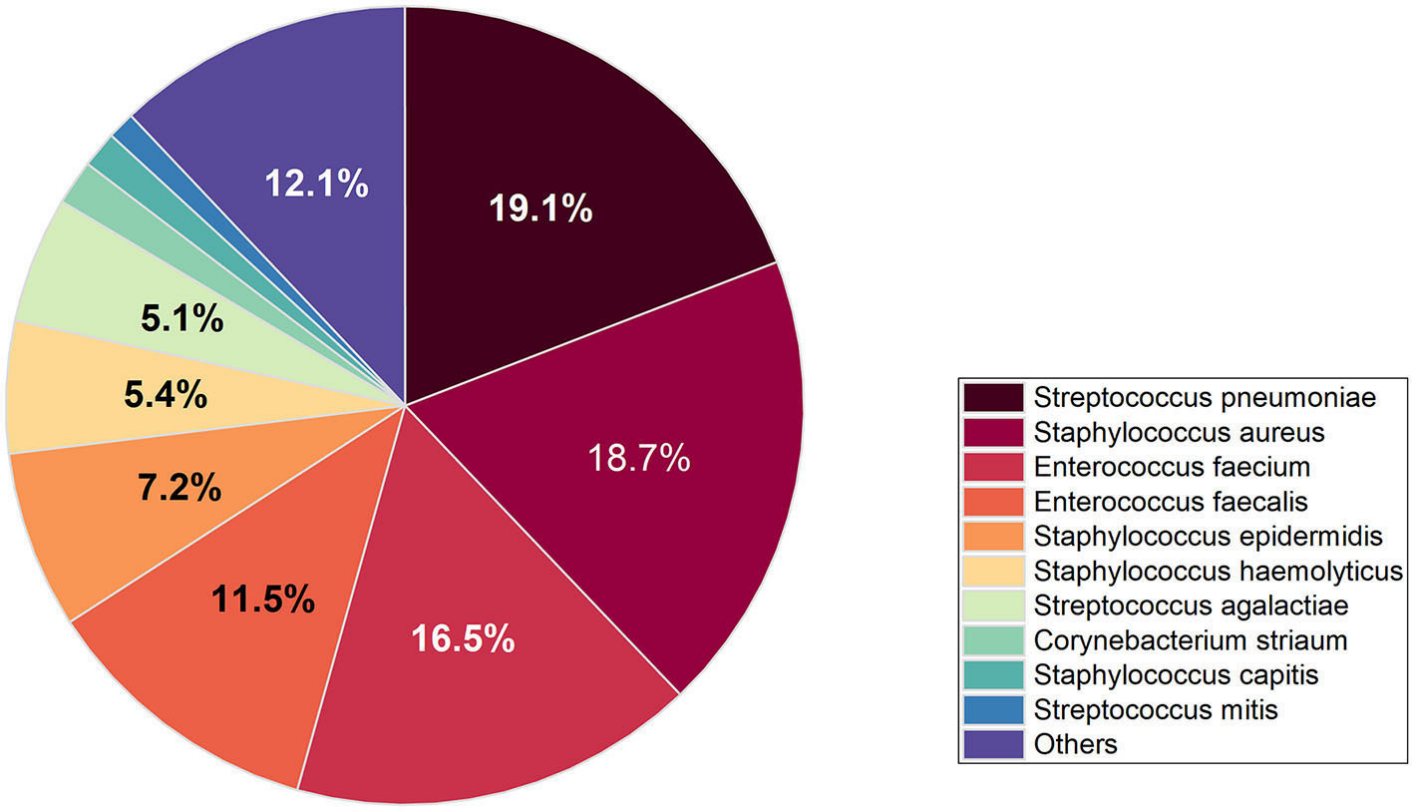
Distribution of Gram-positive bacteria strains in the year of 2021.

**Figure 2:**
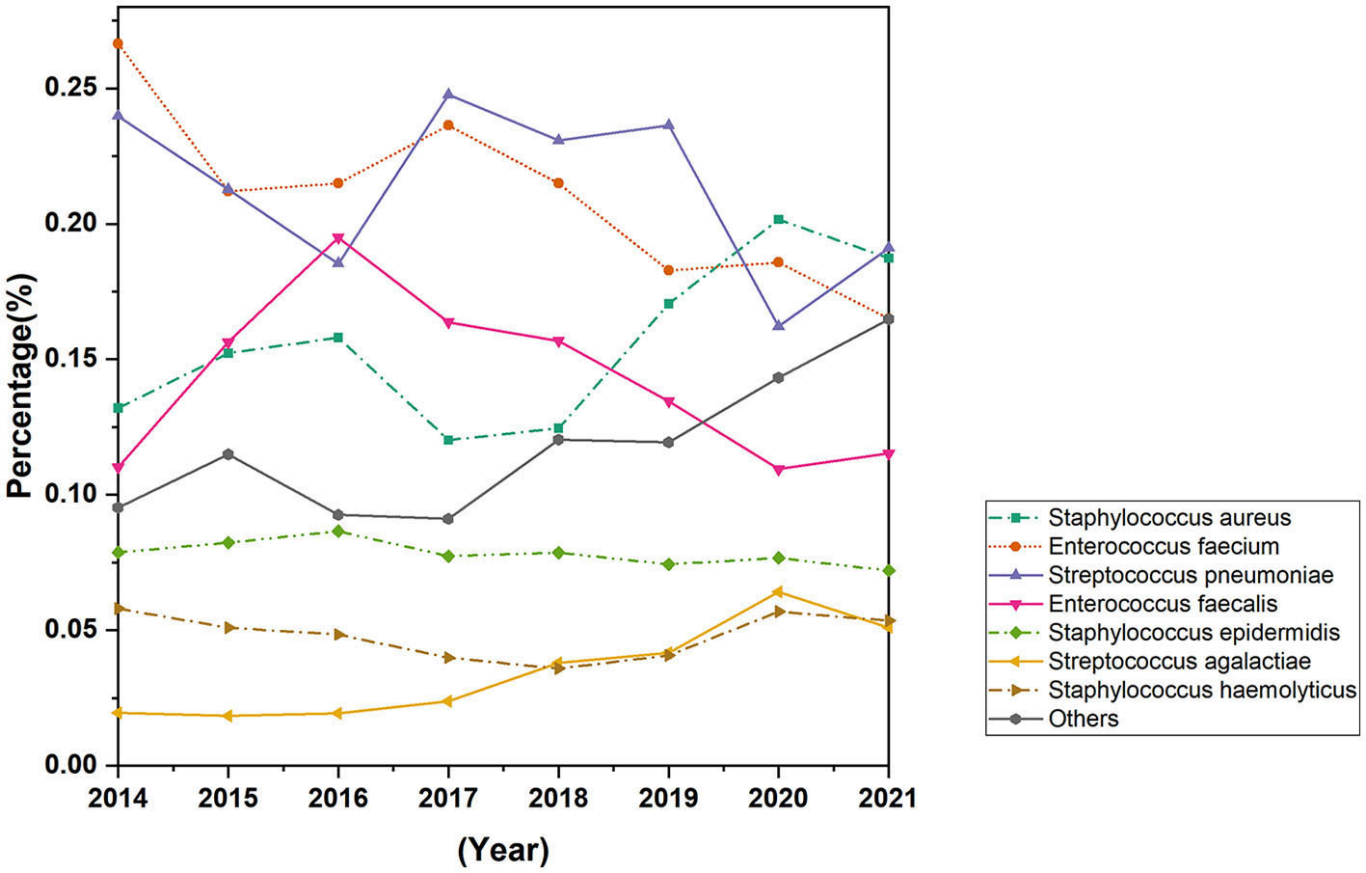
Trends in distribution rates of most common Gram-positive bacteria isolated from 2014 to 2021.

The study encompassed all Gram-positive bacteria strains isolated from patients who had presented to the hospital over an 8-year period spanning from 2014 to 2021, 94.8% of the strains were isolated from hospitalized patients and 5.2% of strains were isolated from outpatients. A total of 15,217 strains of Gram-positive bacteria were isolated from 56.6% (8,613) male patients and 43.4% (6,604) female patients. The increase of Gram-positive isolates showed no variation in terms of gender and age. However, the isolation rate among male patients was observed to be higher than that among female patients, ranged from 54 to 58% in men and from 42 to 46% in women. The total number of Gram-positive bacteria detected increased from 1,689 in 2014 to 2,429 in 2021. The seven most common species were *Streptococcus pneumoniae* (21.2%), *Staphylococcus aureus* (15.9%), *Enterococcus faecium* (20.6%), *Enterococcus faecalis* (14.0%), and *Staphylococcus epidermidis* (7.8%), *Staphylococcus haemolyticus* (4.8%), *Streptococcus agalactiae* (3.6%). The distribution of Gram-positive bacteria strains in the year of 2021 was showed in Figure 1. The isolation rates of *Staphylococcus aureus* and *Streptococcus agalactiae* showed increasing trends during the 8 year-period, while that of *Enterococcus faecium* and *Enterococcus faecalis* decreased in recent years (Figure 2). Except for the most common seven bacteria species, the separation rates of other bacteria species isolated from the clinic experienced an increasing trend. The isolation of *Corynebacterium striatum*, coagulase-negative staphylococcus such as *Staphylococcus hominis* and *Staphylococcus capitis*, enterococcus such as *Enterococcus gallinarum* and *Enterococcus casselifavus*, and α-hemolytic streptococcus such as *Streptococcus mitis* and *Streptococcus angina* have been on the rise, and most of these strains were isolated from critical patients. In the past 8 years, the number of species increased from 38 in 2014 to 60 in 2021, more and more less common Gram-positive bacilli such as *Acanthobacter acidophilus, Bacillus cereus* and *Bacillus pumilus* have been isolated.

### *Staphylococcus aureus* and *Staphylococcus epidermidis*

**Figure 3:**
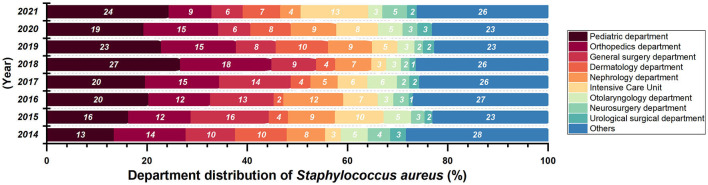
Department distribution of *Staphylococcus aureus* isolated from 2014 to 2021.

Most of the *Staphylococcus aureus* were isolated form Surgery Department and Pediatric Department (Figure 3) from 2014 to 2018. There are 2412 collected *Staphylococcus aureus* strains, and the isolation rate of *S. aureus* in General Surgery Department (*P* = 0.01874, *Z* = −2.3506) had a significant decrease. Isolates were mostly collected from secretion samples (912 isolates, 38.0%), followed by sputum (555 isolates, 23.1%) and blood (392 isolates, 16.3%).

**Figure 4:**
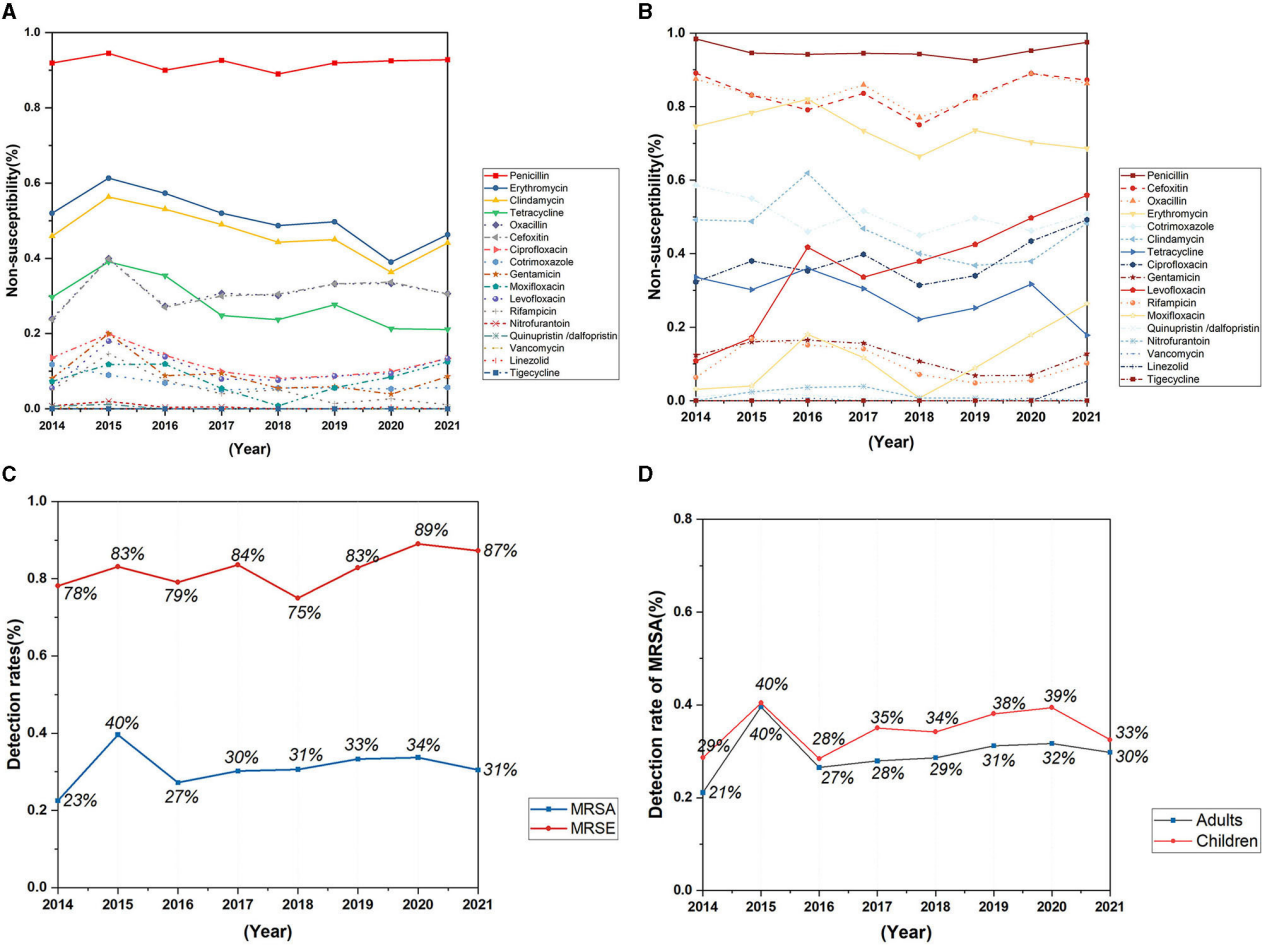
Trends in antimicrobial non-susceptibility of *Staphylococcus aureus* and *Staphylococcus epidermidis* from 2014 to 2021. **(A)** Trends in antimicrobial non-susceptibility of *Staphylococcus aureus*; **(B)** trends in antimicrobial non-susceptibility of *Staphylococcus epidermidis*; **(C)** trends in detection rates of MRSA and MRSE; **(D)** trends in detection rates of MRSA in adults and children.

The detection rate of Methicillin-resistant *Staphylococcus aureus* (MRSA) has not changed significantly in recent years, with a detection rate of 30.5% in 2021 (Figure 4C). The replacement test of relevant samples based on time pairing showed a significant difference in the detection rate of MRSA in children and adults (*P* = 0.0087, *Z* = −2.5205). The detection rate of MRSA in children was higher than that in adults, and the detection rate of MRSA in 2021 was 32.5% in children and 29.8% in adults (Figure 4D). The resistance rates of *Staphylococcus aureus* to penicillin, linezolid, and tigecycline did not change significantly during the past 8 years, but the resistance rates to erythromycin, tetracycline, clindamycin, rifampicin, and nitrofurantoin showed a decreasing trend, and the difference was statistically significant (Table 1). No vancomycin-resistant *Staphylococcus aureus* (VRSA) and vancomycin-intermediate *Staphylococcus aureus* (VISA) was found in the isolates.

**Table 1:** *P* and *Z* value of Manner-Kendall test of the resistance rates in *Staphylococcus Aureus* and *Staphylococcus Epidermidis* over the past 8 years.

	***Staphylococcus aureus***		***Staphylococcus epidermidis***	
	***P***	***Z***	***P***	***Z***
Penicillin	NS	0.249	NS	−0.1237
Erythromycin	0.0248	−2.244	NS	−1.6083
Clindamycin	0.0350	−2.100	NS	−1.6083
Tetracycline	0.0190	−2.350	NS	1.3609
Oxacillin	NS	0.866	NS	0.1237
Cefoxitin	NS	1.361	NS	0.0000
Ciprofloxacin	NS	−0.997	NS	1.3609
Cotrimoxazole	NS	−1.361	NS	−1.1135
Gentamicin	NS	−1.1135	NS	−1.1135
Moxifloxacin	NS	0.3711	NS	1.3609
Levofloxacin	NS	0.3711	0.0044	2.8455
Rifampicin	0.0350	−2.1030	NS	−1.1135
Nitrofurantoin	0.0239	−2.2583	NS	−1.0187
Quinupristin/dalfopristin	NS	−1.6440	NS	−1.5281
Vancomycin	NS	0.0000	NS	0.0000
Linezolid	NS	0.8730	NS	1.3093
Tigecycline	NS	0.0000	NS	0.0000

NS, not significant.

A total of 1,151 *Staphylococcus epidermidis* were mainly collected from blood (520 isolates, 45.2%), secrection (169 isolates, 14.7%), and urine (138 isolates, 12.0%), and most of the isolates were came from the departments of pediatrics, general surgery, urology, nephrology and ICU, but no significant change of the isolation rates of each department was found over the years. *Staphylococcus epidermidis* was more resistant than *Staphylococcus aureus* (Figures 4A, B), and the resistance rates to levofloxacin increased significantly, with the resistance rate reaching 55.9% in 2021 (Figure 4B), the detection rate of Methicillin-resistant *Staphylococcus epidermidis* (MRSE) was 87% in 2021 (Figure 4C).

### *Enterococcus faecium* and *Enterococcus faecalis*

*Enterococcus faecium* and *Enterococcus faecalis* were more frequently found in urine as well as bile and abdominal fluid. *Enterococcus faecium* was mainly collected from General surgery Department, Intensive Care Unit, Urological surgical Department, Pediatric Department, and Nephrology Department, and most of the *Enterococcus faecalis* was isolated from General Surgery Department, Urological Surgical Department, Orthopedics Department, Intensive Care Unit, and Nephrology Department.

**Figure 5:**
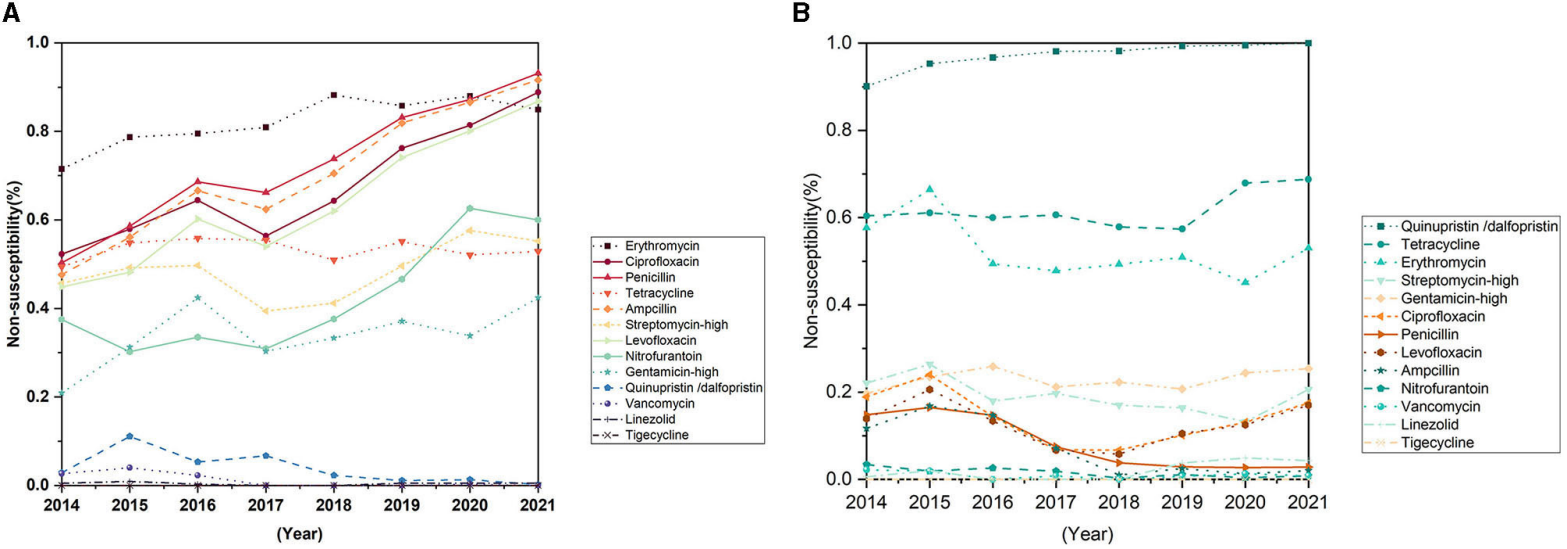
Trends in antimicrobial non-susceptibility of *Enterococcus faecium* and *Enterococcus faecalis* from 2014 to 2021. **(A)** Trends in antimicrobial non-susceptibility of *Enterococcus faecium*. **(B)** Trends in antimicrobial non-susceptibility of *Enterococcus faecalis*.

*Enterococcus faecium* is more resistant against the antibiotics than *Enterococcus faecalis* (Figures 5A, B). During the past 8 years, the resistance rates of *Enterococcus faecium* to penicillin, ampicillin, and the quinolone antibiotics, such as ciprofloxacin and levofloxacin increased significantly, reaching 93.1, 91.6, 86.8 and 86.8% respectively in the year of 2021 (Figure 5B; Table 2). The rate of resistance to nitrofurantoin increased gradually from 37.5% in 2014 to 60.0% in 2021. In addition, the resistance rate to erythromycin rose slowly from 2014 and remained at a high level in recent years, reaching 84.9% in 2021. Although, the resistance rate to quinupristin/dalfopristin decreased significantly over the past 8 years. *Enterococcus faecium* was susceptible to vancomycin. Most of the resistance rates to antibiotics of *Enterococcus faecalis* did not change significantly, except for that of the penicillin experienced a significant decrease from 14.8% in 2014 to 2.8% in 2021.

**Table 2:** *P* and *Z* value of Manner-Kendall test of the resistance rates in *Enterococcus Faecium* and *Enterococcus Faecalis* over the past 8 years.

	***Enterococcus faecium***		***Enterococcus faecalis***	
	***P***	***Z***	***P***	***Z***
Erythromycin	0.0355	2.1032	NS	−0.8660
Ciprofloxacin	0.0094	2.5981	NS	−0.6186
Penicillin	0.0020	3.0929	0.0044	−2.8455
Tetracycline	NS	0.000	NS	0.37115
Ampcillin	0.0020	3.0929	NS	−1.8558
Streptomycin-high	NS	1.3609	NS	−1.6083
Levofloxacin	0.0020	3.0929	NS	−0.3711
Nitrofurantoin	0.0355	2.1032	NS	−1.8558
Gentamicin-high	NS	1.8558	NS	0.8660
Quinupristin/dalfopristin	0.0355	−2.1032	0.0008	3.3404
Vancomycin	NS	−1.7269	NS	−0.4987
Linezolid	NS	0.0000	NS	1.2734
Tigecycline	1	0.0000	1	0.0000

NS, not significant.

### Streptococcus pneumoniae

Most of the *Streptococcus pneumoniae* were isolated from respiratory specimens such as sputum (2,777 isolates, 86.5%) and bronchoalveolar lavage fluid (249 isolates, 7.8%), 7 strains were collected from cerebrospinal fluid. Of these samples, 2,974 (92.6%) strains were collected from Pediatric Department, and 50 (1.6%) strains were isolated from Pneumology Department.

**Figure 6:**
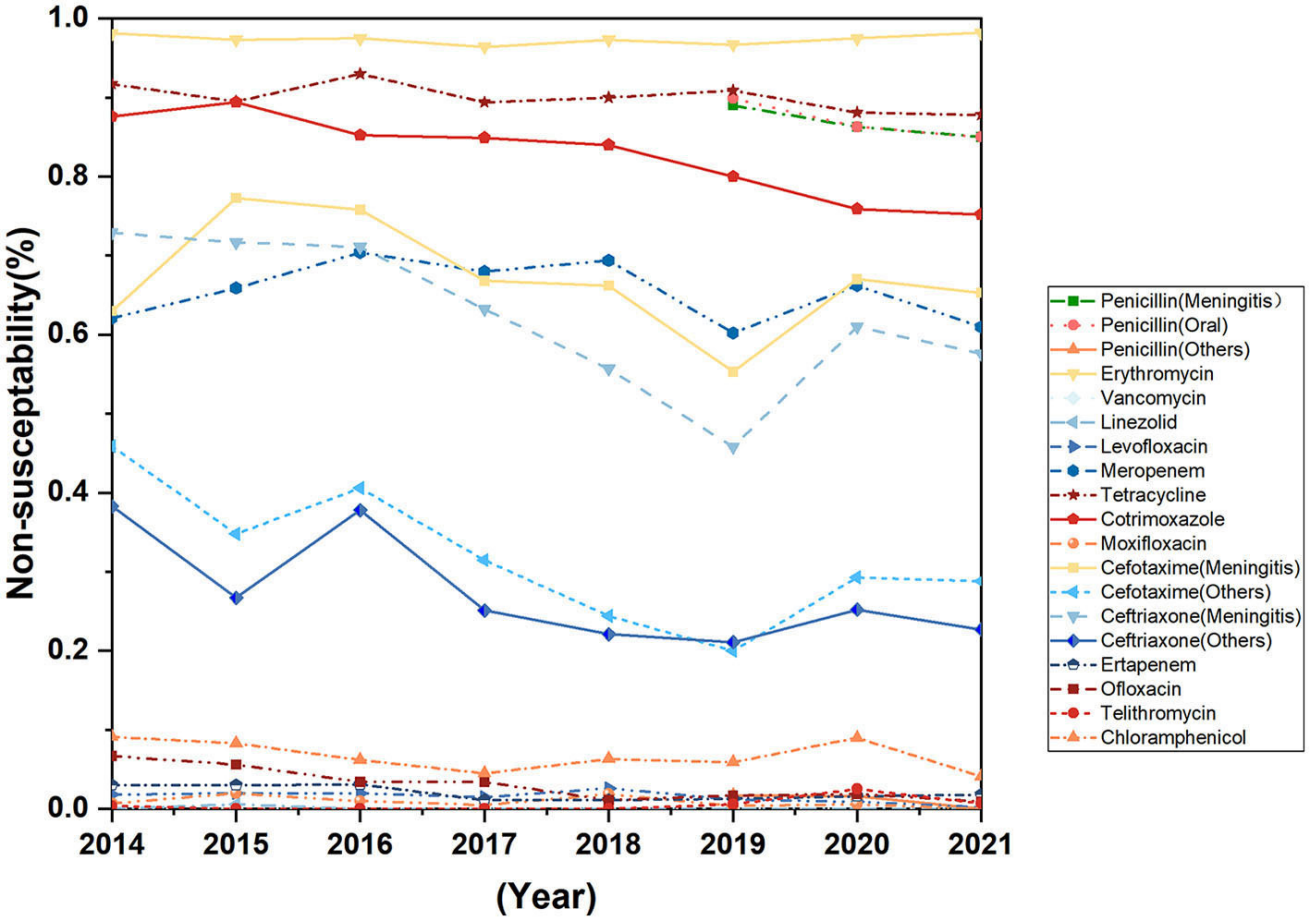
Trends in antimicrobial non-susceptibility of *Streptococcus pneumoniae* from 2014 to 2021.

The resistance rates of *Streptococcus pneumoniae* against cefotaxime (non-meningitis), ceftriaxone (meningitis), cotrimoxazole and ofloxacin decreased during the past 8 years, the difference was statistically significant. *Streptococcus pneumoniae* is highly resistant to erythromycin, tetracycline, and Cotrimoxazole (Figure 6). None of the seven strains of *Streptococcus pneumoniae* isolated from cerebrospinal fluid were susceptible to erythromycin, meropenem, ceftriaxone, and penicillin, but all strains were susceptible to vancomycin, linezolid, levofloxacin, moxifloxacin, and telomycin (Table 3).

**Table 3:** Drug resistance of *Streptococcus pneumoniae* isolated from cerebrospinal fluid.

**Antibiotics**	**Detected isolates**	**R**	**I**	**S**	**Resistance rate**
Penicillin	7	6	1	0	100.0%
Erythromycin	7	7	0	0	100.0%
Vancomycin	7	0	0	7	0.0%
Linezolid	7	0	0	7	0.0%
Levofloxacin	7	0	0	7	0.0%
Meropenem	7	2	5	0	100.0%
Tetracycline	7	5	0	2	71.4%
Cotrimoxazole	7	6	0	1	85.7%
Moxifloxacin	7	0	0	7	0.0%
Cefotaxime	7	1	5	1	85.7%
Ceftriaxone	7	2	5	0	100.0%
Ertapenem	7	1	0	6	14.30%
Ofloxacin	7	1	0	6	14.30%
Telithromycin	7	0	0	7	0.0%
Chloramphenicol	7	1	0	6	14.30%

### Streptococcus agalactiae

The isolation rate of *Streptococcus agalactiae* increased gradually, from 2.0% in 2014 to 5.1% in 2021, in synch with the isolation rate of strains isolated from non-pregnant patients. In the year of 2021, 92.4% of *Streptococcus agalactiae* was isolated from non-pregnant patients. *Streptococcus agalactiae* was mainly isolated from female patients (78.5%). Eighty-seven strains were isolated from pregnant women and 12 strains were isolated from infants. Most of the samples were separated from genital secretions (65.6%), urine (22.9%) and blood (5.8%). *Streptococcus agalactiae* was sensitive to penicillin, ampicillin, tigecycline, linezolid and vancomycin, with the sensitivity rates of 100.0% in 2021. The resistance rates to levofloxacin and moxifloxacin decreased obviously, from 57.60 and 84.90% in 2014 to 26.8 and 26.9% in 2021, respectively. And the rate of resistance to levofloxacin was significantly lower than the national level of 47.9% in 2021. The resistance rate of clindamycin went up from 81.9% and remained at a high level since 2014, the resistance rate of 2021 is 99.2%, which is significantly higher than the national level of 59.7% (Hu Fupin et al., 2022).

## Discussion

This study showed that during the 8-year period from 2014 to 2021, the total number and the species of Gram-positive bacteria isolated from clinic increased continuously, the isolation rates of *Staphylococcus aureus* and *Streptococcus agalactiae* increased gradually, while the isolation rates of *Enterococcus faecium* and *Enterococcus faecalis* decreased. The isolation rates of some rarely detected Gram-positive opportunistic pathogens increased in critical patients.

*Staphylococcus aureus* infections are common, and can be associated with severe clinical manifestations. The *Staphylococcus aureus* infections related clinical disease spectrum is constantly changing (Tong et al., 2015). In this study, *S. aureus* was mainly isolated from secretions, lower respiratory tract, and blood samples. The reason may be that *S. aureus* can be isolated from the human nasal mucosal symbiotic flora of 20–40% of the general population. When the skin and mucosal barriers are damaged, for example, due to chronic skin conditions, wounds, or surgical interventions, *Staphylococcus aureus* can enter underlying tissues or bloodstream and then cause infection (Rodríguez-Lázaro et al., 2017; Lakhundi and Zhang, 2018). Therefore, besides optimizing the medication regimen, it is also very important to help *Staphylococcus aureus* susceptible population to reduce the incidence of infection. In this study, the proportion of *Staphylococcus aureus* detected in general surgery decreased significantly during the past 8 years, which may be related to the continuous improvement of wound management in general surgery patients. No significant change was observed in the detection rate of MRSA in recent years, and the detection rate of MRSA in 2021 was comparable to the national detection rate of 30.5% (Hu Fupin et al., 2022). The detection rate of MRSA in children was slightly higher than that in adults, and the MRSA separation rate of children in this hospital is higher than the national level. This may not only because children have fewer antibiotic options than adults, and are more susceptible to bacterial resistance and MRSA prevalence, but also because the pediatric department of the hospital in this study has larger scale compared with other departments, and has more pediatric intensive care unit beds than other general hospitals. In this study, the resistance rates of *Staphylococcus aureus* to erythromycin, clindamycin, tetracycline and rifampicin decreased, and the difference was statistically significant. No vancomycin resistant *Staphylococcus aureus* strain was isolated.

**Figure 7:**
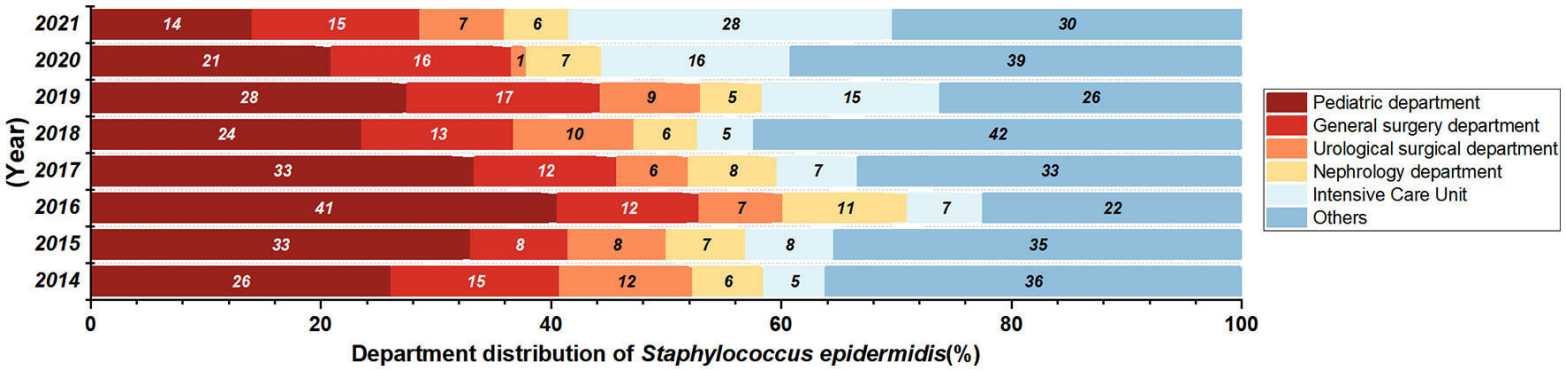
Department distribution of *Staphylococcus epidermidis* isolated from 2014 to 2021.

*Staphylococcus epidermidis* is an important member of the human microbiome and is widely found on healthy skin. In this study, the strains were mostly isolated from the departments of pediatrics, general surgery and Intensive Care Unit (Figure 7), and most of the *Staphylococcus epidermidis* in pediatrics were isolated from children with leukemia and pneumonia, while general surgery and ICU samples were mainly isolated from blood or central venous catheterization samples from patients undergoing hepatobiliary surgery and patients with acute pancreatitis. *Staphylococcus epidermidis* were also isolated from departments of patients with compromised immunity or requiring central venous catheterization, such as department of hematology and nephrology, indicating that patients with low immunity and long-term antibiotic use are more susceptible to *Staphylococcus epidermidis* infection, and the improvement of the ward environment and management of central vein catheterization of susceptible patients are very important. The levofloxacin resistance rate of *Staphylococcus epidermidis* increased significantly, while the ciprofloxacin resistance rate increased, and no significant difference was observed statistically. According to the study of Høiby et al. (1997), extensive use of quinolone antibiotics and continuous excretion from sweat into the skin can increase the resistance of *Staphylococcus epidermidis* to quinolone antibiotics, and the increased resistance rates to levofloxacin and ciprofloxacin in this study may be related to this mechanism. Previous studies have shown that rifampicin-resistant *Staphylococcus epidermidis* strains are more likely to develop resistance to a variety of other antibiotics, and certain rpoB mutant strains have been shown to have heterogeneous resistance to vancomycin (Zalewska et al., 2021). In this study, no significant change was showed in the resistance rate of *Staphylococcus epidermidis* to rifampicin in the past 8 years. However, it should be noticed that the resistance rate to rifampicin reached 10.2% in 2021, higher than the national level (8.7%) and the level of some regions in Europe (8.9%) (Zalewska et al., 2021; Hu Fupin et al., 2022).

Enterococcus is an opportunistic pathogen and an important pathogen of nosocomial infection. Its infection mainly has underlying diseases and susceptibility factors (Zhao et al., 2021). Globally, enterococcus bacteria are the major cause of health care-associated infections (HAIs) in the urinary tract, soft tissue and implanted medical devices (García-Solache and Rice, 2019). The main susceptibility factors of enterococcus were broad-spectrum antibiotic exposure, ICU admission and invasive operation. In this study, the isolates were mainly from the department of general surgery, ICU and department of urological surgery, the proportion of isolates in ICU was significantly increased in the 8-year period, and the proportion of enterococcus isolated from drainage samples was significantly increased. The resistance rates of *Enterococcus faecium* increased significantly in the past 8 years, and the resistance rates to penicillin, ampicillin, erythromycin, levofloxacin, ciprofloxacin and furantoin rose rapidly from 50.3, 47.6, 71.5, 44.9, 52.3, and 37.5% in 2014 to 91.6, 84.9, 86.8, 86.8, 60.0, 93.1% in 2021, respectively. Although the change of high-level gentamicin resistance rate was not statistically significant, it also showed an increasing trend (*P* = 0.063), which further reduced the selection of antibiotics in clinical practice. The reason for this may be related to *Enterococcus faecium*'s long-term antibiotic exposure and its surprising adaptability to harsh environments, which include remarkable genomic plasticity, the ability to evade the immune system, the ability to attach to host cells, extracellular matrix (EM) and insert materials (such as various medical devices), the ability to form biofilms, and the ability to tolerate disinfectants, making them more resistant to antibiotic killing and phagocytic attacks (Mohamed and Huang, 2007; García-Solache and Rice, 2019). There was no significant change in the resistance rate of *Enterococcus faecium* to tetracycline, while the resistance rate of *Enterococcus faecium* to quinupristin/dalfopristin decreased, the reason of decreasing may be related to the less clinical use of tetracycline and quinupristin/dalfopristin in recent years. The resistance rate of *Enterococcus faecalis* to most of the tested antibiotics was significantly lower than that of *Enterococcus faecium*, and the resistance rates to most antibiotics did not change significantly over the past 8 years. The resistance rate of *Enterococcus faecalis* to penicillin decreased significantly, and the resistance rate to ampicillin showed a downward trend, but there was no statistical significance (*P* = 0.063). Currently, monotherapy with vancomycin or vancomycin combined with linezolid and other antibiotics are the preferred clinical treatment for multidrug-resistant enterococcal infections requiring bactericidal treatment. In this study, there was no significant change in the overall resistance rate of *Enterococcus faecium* and *Enterococcus faecalis* to vancomycin and linezolid antibiotics, and several vancomycin and linezolid resistant strains were isolated in some years. However, studies have shown that VRE detection is on the rise in some areas (Jahansepas et al., 2018), which raises concerns about whether vancomycin would continue to be the drug of first choice for many of the multidrug-resistant Gram-positive infections in the near future.

*Streptococcus pneumoniae* is widely distributed in nature environment and is present in the nasopharynx of 27–65% of children. It is an important opportunistic pathogen (Zhao et al., 2019) and mainly transmitted by respiratory tract. In our study, the isolation rate of *Streptococcus pneumoniae* decreased significantly in 2020 when COVID-19 pandemic broke out, compared with the year of 2019. Another study conducted in China shows that the total number of influenza tests of a children's hospital decreased by 68.83% and the total number of Mycoplasma pneumoniae tests decreased by 61.58% compared with the pre-epidemic period, and the positive rate of detection also decreased significantly (Min et al, 2022), indicating that a series of prevention and control measures against COVID-19, such as frequent hand-washing and wearing masks, may also effectively prevent the transmission of other respiratory pathogens. In this study, more than 90% of *Streptococcus pneumoniae* were isolated from children, 94.2% were from respiratory tract samples such as sputum and alveolar lavage fluid, and 4.4% were from blood samples. Three different penicillin sensitivity criteria have been applied according to the route of use and type of disease (CLSI, 2010), and there was no significant change in the resistance rate of *Streptococcus pneumoniae* to penicillin (non-meningitis) and penicillin (meningitis) since the criteria was applied to the clinical reports in 2018. The resistance rate to cefepime (non-meningitis) and ceftriaxone (meningitis) decreased significantly during the past 8 years. The difference in ceftriaxone (non-meningitis) resistance rates was not statistically significant, but showed a downward trend, which may be related to the intentional avoidance of the use of these two drugs by clinicians in order to avoid the rise of resistance to these two drugs that can cross the blood-brain barrier. The drug resistance rate to cotrimoxazole decreased, and the difference was statistically significant. The resistance rate to ofloxacin continued to decrease during the past 8 years, to 0.009% in 2021, while the resistance rates to levofloxacin and moxifloxacin remained at a low level, which may be related to the low use of cotrimoxazole and quinolones in the pediatric population (Shi et al., 2020). There is an obvious correlation between antibiotic resistance and the serotype of *Streptococcus pneumoniae*. For example, half of our penicillin-resistant strains have been identified as serotype 19F (Pan et al., 2015), therefore, the effect of vaccine introduction on antibiotic resistance cannot be ruled out. However, due to the high cost of *Streptococcus pneumoniae* vaccine and the low vaccination rate of children in China, the impact of vaccine use on antibiotic sensitivity of *Streptococcus pneumoniae* isolates remains to be evaluated (Shi et al., 2020). The results of this study showed that although the resistance rates of *Streptococcus pneumoniae* to some antibiotics decreased, the resistance rates of *Streptococcus pneumoniae* to β-lactam and macrolide antibiotics were still high, which could resist the standard treatment of these two antibiotics, bringing great challenges to clinical diagnosis and treatment. And furthermore, there are fewer antibiotic choices for children with meningitis (see Table 3). Therefore, as a high burden country with *Streptococcus pneumoniae* infection, while reducing the irrational use of antibiotics, it is also very important for our country to proactively prevent the infection of *Streptococcus pneumoniae* by strengthening the monitoring of the prevalence of serotypes of *Streptococcus pneumoniae*, enhancing vaccine research and development, and improving vaccine coverage of susceptible population.

*Streptococcus agalactiae* or Group B Streptococcus (GBS) is an opportunistic pathogen that causes serious illness in newborns, pregnant women and adults (Tulyaprawat et al., 2021). In recent years, the infection cases of non-pregnant adults caused by GBS have been on the rise, with relatively serious patient conditions and high fatality rate (Jingxian et al., 2022). In our study, the increase in *Streptococcus agalactiae* isolation rate was due in part to the increase in isolation from non-pregnant adult patients. In this study, *Streptococcus agalactiae* was highly sensitive to penicillin and vancomycin, but highly resistant to second-line drugs such as tetracycline, clindamycin and erythromycin. There is a certain correlation between the serum type, ST type and the drug resistance phenotype in *Streptococcus agalactiae*. Study shown that the proportion of serum type III has dropped sharply from nearly 90% to about 50% (Tulyaprawat et al., 2021), indicates that second-line antibiotic resistance may be the selective pressure that leads to the high prevalence of non-serum type III isolates, which may account for the high non-neonatal mortality from GBS infection. The rates of drug resistance to quinolone antibiotics decreased and was lower than the national average level (Hu Fupin et al., 2022), but there was still a certain failure rate of empirical use of quinolone antibiotics in patients with GBS urinary tract infection, and medication should be adjusted according to the results of drug sensitivity test.

In this study, we found that the composition and drug resistance spectrum of clinically isolated Gram-positive bacteria have changed significantly compared with that of 8 years ago. With the continuous improvement of the awareness of severe drug resistance situation and the more reasonable and standardized use of antibiotics, the resistance rates of Gram-positive coccus which are serious threats to patients' lives such as *Staphylococcus aureus* and *Streptococcus pneumoniae*, has decreased. But we observed a very clear trend of rising resistance in *Enterococcus faecium*, which has received relatively little attention. The rise of drug resistance in *Enterococcus faecium* not only poses serious challenges in the diagnosis and treatment of patients with *Enterococcus faecium* infection itself, but also has influence for drug resistance in other strains. For example, vancomycin-resistant *Staphylococcus aureus* (VRSA), which was first identified in the United States in 2002, was believed to get the vancomycin resistance gene located in plasmid transposon Tn1546 transferred from *Enterococcus faecalis* (Weigel et al., 2003). This reminds us that while prioritizing some strains that seriously affect patient outcomes, the drug resistance situation of other strains should not be ignored. At present, there are many bacterial resistance monitoring platforms that update and share real-time antibiotic-resistance-related resistome data, such as CARD (the Comprehensive Antibiotic Research Database), which describes the molecular basis of antibiotic resistance in detail, including known determinants of AMR (i.e., acquired resistance genes, resistance mutations of household genes, efflux overexpression), drug targets, and molecular mechanisms of drug resistance (Alcock et al., 2020), provides a powerful platform for antibiotic resistome surveillance. On this basis, more attention should be paid to the changes in drug resistance among different species, so as to timely detect changes in drug resistance that are not easily detected, and to seek and develop comprehensive and multi-pronged strategies for the prevention and control of drug-resistant bacteria.

## Data availability statement

The original contributions presented in the study are included in the article/supplementary material, further inquiries can be directed to the corresponding author.

## Ethics statement

The studies involving humans were approved by Ethics Committee of Hunan Provincial People's Hospital. The studies were conducted in accordance with the local legislation and institutional requirements. Written informed consent for participation was not required from the participants or the participants' legal guardians/next of kin because To quote from the International Ethical Guidelines for Biomedical Research, an Ethics Committee may waive informed consent if the study design involves minimal harm and the requirement to sign informed consent would make it impossible to conduct the study.

## Author contributions

LX and XZ conceived and designed the study. XZ, LX, LT, and TS contributed to data collection and experiments implementation. XZ, LX, LT, PO, HM, JP, and TS contributed to data analysis and data interpretation. XZ and LX contributed to writing the report and revising the report. All authors read and approved the final version of the manuscript.
